# The CENP-B homolog, Abp1, interacts with the initiation protein Cdc23 (MCM10) and is required for efficient DNA replication in fission yeast

**DOI:** 10.1186/1747-1028-1-27

**Published:** 2006-11-17

**Authors:** Alexandra M Locovei, Maria-Grazia Spiga, Katsunori Tanaka, Yota Murakami, Gennaro D'Urso

**Affiliations:** 1 University of Miami School of Medicine, Department of Molecular and Cellular Pharmacology, P.O. Box 016189, Miami, FL, 33101, USA; 2 Department of Applied Bioscience and Biotechnology, Faculty of Life and Environmental Science, Shimane University, Matsue, 690-8504, Shimane, Japan; 3 Institute for Virus Research, Kyoto University, Sakyo-ku, Kyoto, 606-8507, Japan

## Abstract

Abp1, and the closely related Cbh1 and Cbh2 are homologous to the human centromere-binding protein CENP-B that has been implicated in the assembly of centromeric heterochromatin. Fission yeast cells lacking Abp1 show an increase in mini-chromosome instability suggesting that Abp1 is important for chromosome segregation and/or DNA synthesis. Here we show that Abp1 interacts with the DNA replication protein Cdc23 (MCM10) in a two-hybrid assay, and that the *Δabp1 *mutant displays a synthetic phenotype with a *cdc23 *temperature-sensitive mutant. Moreover, genetic interactions were also observed between *abp1*^+ ^and four additional DNA replication initiation genes *cdc18*^+^, *cdc21*^+^, *orc1*^+^, and *orc2*^+^. Interestingly, we find that S phase is delayed in cells deleted for *abp1*^+ ^when released from a G1 block. However, no delay is observed when cells are released from an early S phase arrest induced by hydroxyurea suggesting that Abp1 functions prior to, or coincident with, the initiation of DNA replication.

## Background

DNA replication requires the assembly of replicative complexes (RCs) at chromosomal replication origins. The first step in this process involves assembly of pre-RCs in early G1 [1-6]. Formation of these complexes is restricted to a period of the cell cycle following conclusion of mitosis when cyclin-dependent kinase (Cdk) activity is low [7-9]. Subsequent conversion of the pre-RC to an active replicative complex at the beginning of S phase is dependent on re-activation of Cdk activity that leads to the recruitment of essential replication proteins to origin DNA. This mechanism ensures that cells replicate once and only once during each cell division cycle [10]. Unlike simple organisms like bacteria, yeast requires that initiation of DNA replication occur on chromatin-bound templates. Although the details of how replication occurs on chromatin are poorly understood, it is possible that remodeling activities that promote protein-protein and protein-DNA interactions on chromatin are important to allow efficient replication of these templates [11-13].

Two proteins that are believed to be required for assembly of pre-RCs in fission yeast are Cdc18 (Cdc6) and Cdt1 (Cdt1) [14-18]. For clarity, all *S. pombe *gene/protein names will be followed by the corresponding *S. cerevisiae *gene/protein name in parentheses, where a clear homolog exists. These proteins facilitate the loading of Mini-Chromosome-Maintenance (MCMs) proteins to origin DNA in early G1 [19-21]. The Mcm2-7 complex is believed to function as the DNA replicative helicase that unwinds origin DNA at the start of S phase [22-24]. Two additional proteins, Sna41 (Cdc45) and Cdc23 (Mcm10) interact both physically and genetically with components of the initiation complex, and are believed to be important for recruitment of DNA polymerases to the origin-associated pre-RC [25-31].

The *cdc23 (MCM10) *mutant of *S. pombe *was originally identified as a cell cycle mutant defective in the completion of DNA synthesis and was later shown to block in early S phase [28,32]. Subsequent cloning and characterization of the *cdc23 (MCM10) *gene demonstrated that it is homologous to *S. cerevisiae MCM10*, and was capable of rescuing the budding yeast *mcm10 *mutant, *dna43-1 *[33]. In *S. cerevisiae*, Mcm10 binds to replication origins during G1 and S phase suggesting it plays a critical role in both initiation and elongation of DNA replication [29]. However, in fission yeast it is not yet clear whether Cdc23 (Mcm10) binds origins in a cell cycle dependent manner [34]. More recently, Cdc23 (Mcm10) has been shown to interact directly with DNA polymerase α/primase and to stimulate primase activity *in vitro *[29,35]. Cdc23 (Mcm10) has been shown to genetically and physically interact with components of the pre-RC, including Mcm2, 4, 5 and 6 and to facilitate chromatin binding of Cdc45 [28,37]. Two-hybrid analysis also shows that Cdc23 (Mcm10) can interact with Orc1, 2, 5, and 6 [28,36]. These data strongly support a role for Cdc23 (Mcm10) in the initiation of DNA replication. Also, biochemical analysis of purified Cdc23 (Mcm10) protein demonstrates that it is required for efficient phosphorylation of the Mcm2-7 complex by Dfp1-Hsk1 (Dbf4-Cdc7) kinase *in vitro*, and that Cdc23 (Mcm10) can directly interact with Dfp1-Hsk1 (Dbf4-Cdc7) [37].

Ars binding protein 1 (Abp1) was first identified in a search for fission yeast proteins that could retard an ARS (Autonomously Replicating Sequence)-containing DNA fragment in a gel-shift mobility assay [38]. Although Abp1 was shown to be non-essential [39], the protein could bind very tightly to ARS elements *in vitro*. Independently, Abp1 was also identified as a protein that could bind centromeric DNA sequences [39]. These regions of DNA typically contain high concentrations of ARS-related sequences (Takahashi et al, 1992). Consistent with a role in either DNA replication or chromosome segregation deletion of *abp1*^+ ^was shown to decrease mini-chromosome stability [39]. When carefully analyzed, many of the cells deleted for *abp1*^+ ^displayed segregation defects suggesting that Abp1's primary function may be to ensure proper chromosome segregation at the conclusion of mitosis [39]. However, these observations do not rule out the possibility that Abp1 has a role in DNA replication. Moreover, two additional *S. pombe *Abp1-related proteins, called Cbh1 and Cbh2, have been identified and like Abp1, both were shown to be non-essential for viability [40-42]. However deletion of both Abp1 and Cbh1 lead to loss of viability and dramatic morphological changes, including branching and cell elongation. Therefore the function of these proteins is likely to be redundant and essential for normal cell cycle progression [42,43]. More recently, Abp1 has been shown to bind directly to the outer repeats of the *S. pombe *centromere, promoting specific histone modifications that lead to the recruitment of Swi6 and gene silencing [44]. Although currently there is no evidence to suggest that either Abp1 or Swi6 interact with other regions of the genome apart from centromeres and telomeres, it is possible that they interact specifically with replication origin DNA and that their presence at these sites regulate initiation of DNA replication.

Using a two-hybrid system, we have identified Abp1 as a protein that interacts with Cdc23 (Mcm10). Genetic interactions between a *cdc23 (mcm10) *temperature-sensitive mutant and the *Δabp1 *strain provide further support that these two proteins functionally interact. We also show that deletion of *abp1*^+ ^results in a delay in S phase when released from a G1 block consistent with Abp1 having a role in DNA replication initiation.

## Results

### A two-hybrid screen identifies Abp1 as a protein interacting with Cdc23

**Figure 1:**
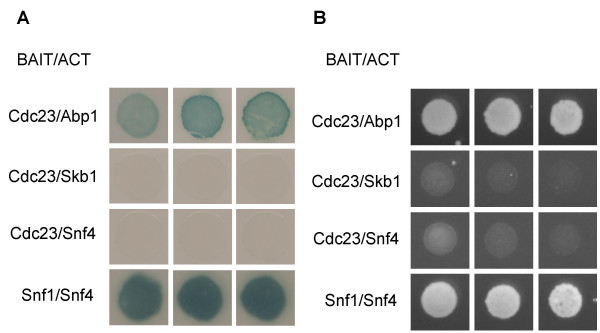
Cdc23 physically interacts with Abp1 in a yeast two-hybrid cDNA library screen. Row 1: Cdc23-Abp1 interaction; rows2, 3: negative controls; row 4: Snf1/Snf4 positive control. **(A)**. β-galactosidase assay. **(B)**. *HIS3 *expression in the presence of 50 mM 3-AT. All experiments shown in triplicate.

To provide additional insights into how Cdc23 (Mcm10) might function during DNA replication initiation, we conducted a two-hybrid interaction screen to identify cDNAs encoding proteins that interact with Cdc23 (Mcm10). One of the proteins identified several times in our screen was ARS-binding protein 1 (Abp1), a protein previously shown to interact with both replication origins and centromere-associated DNA sequence elements. Cdc23 (Mcm10) fused to the DNA binding domain of Gal4 was able to activate lacZ expression from the GAL1 promoter when co-expressed with Abp1 fused to the Gal4 activation domain (Fig. 1A, row 1). As a negative control, when Abp1 was replaced with either Skb1 or Snf4 no LacZ expression was observed (Figure 1A, rows 2 and 3). The two-hybrid interaction between Snf1 and Snf4 is shown as a positive control (Figure 1A, row 4). These two transcription factors have been previously shown to interact using the two-hybrid assay (Durfee et al, 1993).

Consistent with the lacZ data, Cdc23 (Mcm10) fused to the DNA binding domain of Gal4 activated HIS3 under the control of the Gal4 promoter when co-expressed with Abp1 fused to the Gal4 activation domain (Figure 1B, row 1). Co-expression with either Skb1 or Snf4 failed to activate HIS3 (Figure 1B, row 2 and 3) suggesting that the interaction of Cdc23 and Abp1 in the two-hybrid system is specific. As expected, the positive control, Snf1/Snf4 was also able to confer HIS prototrophy (Figure 1B, row 4).

### Genetic interactions between Cdc23 and Abp1

**Figure 2:**
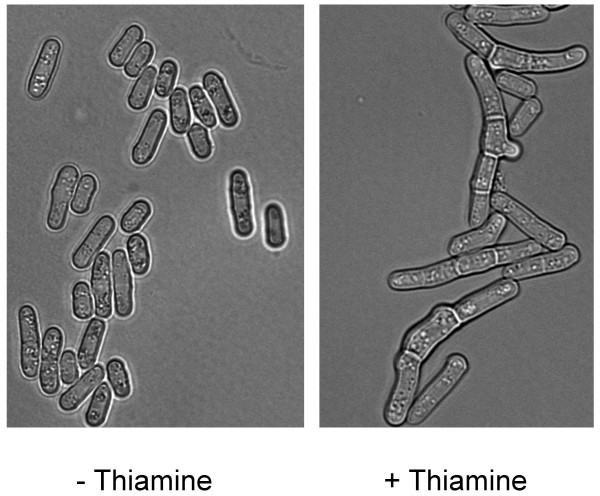
Depletion of Abp1 protein in the triple deletion Δ*abp1 Δcbh1 Δcbh2 int. nmt81-abp1*^+ ^mutant strain leads to cell cycle arrest. Phase contrast microscopy shows cells to be highly elongated 24–36 hrs following addition of 10 μg/μl thiamine (to repress transcription of the *abp1*^+ ^gene) indicating that cell cycle progression is blocked. Cells also show other morphological defects including multi-septation and branching. Left panel: Control (no thiamine). Right panel: Cells after 24 hours of thiamine treatment.

**Figure 3:**
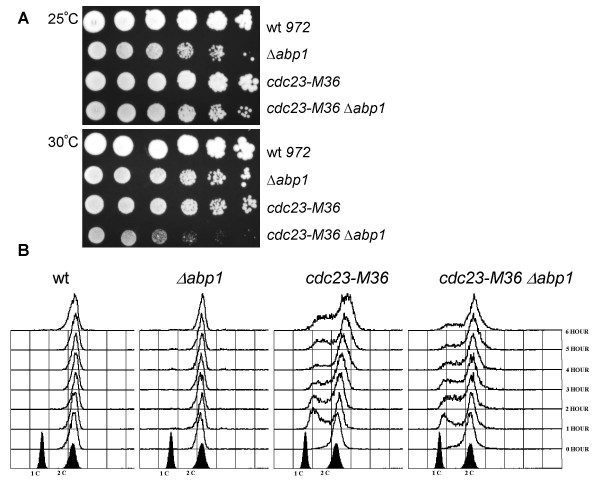
Genetic interactions between *cdc23 *and *abp1*. **(A)**. The double mutant *cdc23-M36 Δabp1 *displays synthetic lethality at 30°C. Serial dilutions of *cdc23-M36, Δabp1 *and *cdc23-M36 Δabp1*, followed by incubation for 4 days at 25°C (upper panel) and 30°C (lower panel). **(B)**. Cell cycle progression profiles of *Δabp1*, *cdc23-M36 *and *cdc23-M36 Δabp1 *at 30°C (from 1–6 hrs) and 25°C at time zero, by flow cytometry anaylsis. Both *cdc23-M36 *and *cdc23-M36 Δabp1 *show G1/S delay.

To further explore potential interactions between Cdc23 (Mcm10) and Abp1, we tested whether mutants defective for Cdc23 (Mcm10) display genetic interactions when crossed to the *Δabp1 *strain. Cells deleted for Abp1 were previously reported to have a slow growth phenotype but otherwise to appear normal. Similarly, Cbh1 or Cbh2 are non-essential for viability (Table 1). However, cells deleted for both *abp1*^+ ^and *cbh1*^+ ^fail to form colonies, suggesting that these proteins provide an essential, albeit redundant, function (data not shown). Cells deleted for *abp1*^+^, *cbh1*^+ ^and *cbh2*^+ ^arrest with a highly elongated and branched terminal morphology [42,43]. We confirmed these results by examining the triple deletion mutant following tetrad analysis (data not shown). In order to better visualize the terminal phenotype of the triple deletion mutant, we constructed a strain deleted for *abp1*^+^, *cbh1*^+^, and *cbh2*^+ ^containing an integrated copy of *abp1*^+ ^under the control of the thiamine repressible *nmt *promoter. When shifted to media containing thiamine (to repress transcription from the *nmt41-abp1*^+ ^gene), this strain appeared highly elongated and branched, indicating that both cell cycle progression and morphology are affected (Figure 2). The *cdc23-M36 *mutant was crossed to the individual mutants *Δabp1*, *Δcbh1 *or *Δcbh2 *and double mutants were isolated. As originally reported, *cdc23-M36 *fails to form colonies at the restrictive temperature of 36°C, but is viable at the intermediate temperature of 30°C (Figure 3A, lower panel, row 3). We found that the double mutant *cdc23-M36 Δabp1 *is less viable then either *cdc23-M36 *or *Δabp1 *when grown at 30°C (Figure 3A, lower panel, row 4), consistent with our two-hybrid data suggesting that Cdc23 (Mcm10) interacts directly with Abp1. We also tested whether the *cdc23-M36 *mutant displays a synthetic phenotype when combined with deletion of either *cbh1 *or *cbh2*, but no genetic interactions were observed (Table 2). Therefore the genetic interaction observed between *cdc23-M36 *and *Δabp1 *appears to be Abp1-specific.

**Table 1:** Phenotype of *abp1*, *cbh1*, and *cbh2 *deletion strains

***S. pombe *strain**	**Growth**	**Phenotype**
*Wild type*	++++	Wild type
Δ*abp1*	++	Wild type
Δ*cbh1*	+++	Wild type
Δ*cbh2*	+++	Wild type
Δ*cbh1Δcbh2*	++	Slightly elongated
Δ*abp1Δcbh2*	+	Slightly elongated
Δ*abp1Δcbh1*	-	Highly elongated/branched, multisepta
Δ*abp1Δcbh1Δcbh2*	-	Highly elongated/branched, multisepta

Note: ++++ = normal growth rate; +++ = slightly slow growth; ++ = slow growth rate; + = very slow growth rate; - = inviable, no growth

**Table 2:** Synthetic Interactions between *Δabp1 *and DNA replication mutants.

***S. pombe *strains**	**Gene product**	**25°C**	**30°C**	**31°C**	**32°C**	**33°C**	**34°C**	**36°C**
*Wild type*		++	++	++	++	++	++	++
Δ*abp1*		+	+	+	+	+	+	+
***cdc23-M36 ***	S phase factor	++	++	++	-	-	-	-
Δ*abp1 cdc23-M36*		+	-	-	-	-	-	-
Δ*abp1Δcbh2*		+	+	+	+	+	+	+
Δ*abp1Δcbh2 cdc23-M36*		+	-	-	-	-	-	-
Δ*cbh1Δcbh2 *		+	+	+	+	+	+	+
Δ*cbh1Δcbh2 cdc23-M36*		+	+	+/-	-	-	-	-
***cdc20-P7***	DNA polymerase	++	++	++	++	++	-	-
Δ*abp1 cdc20-P7*	ε catalytic subunit	+	+	+	+	+	-	-
***cdc20-M10***	DNA polymerase	++	++	++	++	++	+/-	-
Δ*abp1 cdc20-M10*	ε catalytic subunit	+	+	+	+	+	+	-
***cut5-T401***	S phase initiator	++	++	++	++	++	-	-
Δ*abp1 cut5-T401*		+	+	+	+	+	-	-
***cdc30-2H4 ***	ORC complex	++	++	++	+/-	-	-	-
Δ*abp1 cdc30-2H4*		+	+	+/-	-	-	-	-
***cdc18-K46***	S phase initiator	++	++	++	++	++	+	-
Δ*abp1 cdc18-K46*		+	+	+	+	+/-	-	-
***cdc21-M68***	MCM2-7 complex	++	++	++	+	+	-	-
Δ*abp1 cdc21-M68*	subunit 4	+	+	+	+/-	-	-	-
***sna41-912***	S phase initiator	++	++	+/-	-	-	-	-
Δ*abp1 sna41-912*		+	+	+/-	-	-	-	-
***orp2-2***	ORC complex	++	++	++	++	+	-	-
Δ*abp1 orp2-2*		+	+	+	+	+	+	-
***orp2-7 ***	ORC complex	++	++	++	++	++	++	+
Δ*abp1 orp2-7*		+	+	+	+	+	+	-
***cdc1-P13***	DNA polymerase	++	++	-	-	-	-	-
*Δabp1 cdc1-P13*	δ small subunit	+	+	+	-	-	-	-
***cdc6-23***	DNA polymerase	++	-	-	-	-	-	-
*Δabp1 cdc6-23*	δ catalytic subunit	+	+/-	-	-	-	-	-
***cdc27-P11***	DNA polymerase	++	++	++	++	-	-	-
*Δabp1 cdc27-P11*	δ associated factor	+	+	+	+	+/-	-	-
***cdc17-M75***	DNA ligase	++	++	+	+/-	-	-	-
*Δabp1 cdc17-M75*		+	+	+	+	+	+/-	-

Note: ++ = normal growth; + = slow growth; +/- = very slow growth, elongated; = dead, elongated

We then examined the terminal phenotype of either the *cdc23-M36 *mutant alone or the *cdc23-M36 Δabp1 *double mutant following shift to the semipermissive temperature of 30°C. We analyzed DNA content by flow cytometry to determine the precise arrest points for the different strains (Figure 3B). As expected, wildtype or mutant cells grown at the permissive temperature of 25°C display a 2C DNA content, indicating that most cells when growing exponentially are in the G2 phase of the cell cycle. Upon shift to 30°C, *cdc23-M36 Δabp1 *double mutants accumulate with a 1C DNA content indicative of a block to DNA replication initiation. The appearance of this peak is also observed in the *cdc23-M36 *mutant alone, suggesting that depletion of Abp1 does not dramaticall *y *change the arrest point of *cdc23 *mutants, although these cells grow poorly (Figure 3A, lower panel, row 4). Similar to previous reports that show *cdc23 *(*MCM10*) mutants arresting in S phase when shifted to 36°C, *cdc23 *(*MCM10*) mutants grown at 30°C show a pronounced cell cycle delay in early S phase [33]. This is consistent with its proposed role in initiation and further supports the notion that Abp1 interacts with a protein required for DNA replication initiation.

**Figure 4:**
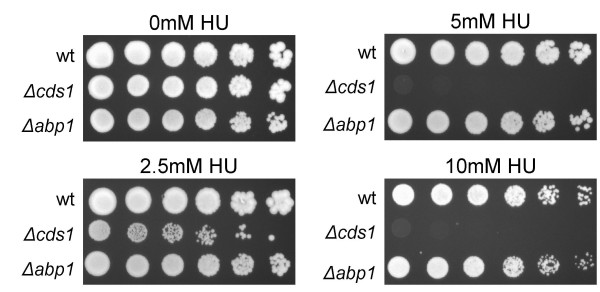
*Δabp1 *is not sensitive to hydroxyurea treatment. Cells were plated on YEA plates containing the indicated concentrations of hydroxyurea (HU) and incubated for 5 days at 32°C. Each row represents a sequential five-fold serial dilution of the initial plating of 10^5 ^cells. The control strain *Δcds1 *looses viability at very low concentrations of HU (2.5 mM) and fails to form colonies at the higher concentrations of 5 and 10 mM HU. Cds1 is essential for the intra-S phase checkpoint activated in response to replication blocks [47]. In contrast, wildtype (wt) or *Δabp1 *display similar sensitivity to HU that is only observed at the highest concentration of 10 mM.

We also tested whether loss of Abp1 displays a synthetic phenotype with other mutants defective in DNA replication. Of the 14 mutants tested, *Δabp1 *showed negative genetic interactions with four additional temperature-sensitive mutants, *orp2-7 (ORC2)*, *cdc30-2H4 (ORC1)*, *cdc18-D46 (CDC6) *and *cdc21-M68 (MCM4)*, all of which encode proteins essential for DNA replication initiation (Table 3). Interestingly, the *orc2-2 *allele was partially suppressed by deletion of *abp1*. On the other hand, no negative synthetic phenotypes were observed between *Δabp1 *and mutants defective in DNA replication elongation, including *cdc1-P13 (POL31)*, *cdc6-23 (POL3)*, *cdc17-M75 (CDC9)*, *cdc27-P11 (POL32)*. However, all of elongation mutants tested were partially suppressed by loss of *abp1 *(see Table 3). It is not yet clear why disruption of *abp1 *might lead to suppression of these mutants. Moreover, the *Δabp1 *strain is not sensitive to treatment with hydroxyurea (Figure 4). Taken together, these results are consistent with Abp1 having a specific role during initiation.

**Table 3:** List of Strains used

*972 h*^-^	P. Nurse
*ade6-704 leu1-32 ura4-D18 h*^-^	P. Nurse
*abp1::ura4*^+^*ade6-704 leu1-32 ura4-D18 h*^-^	L. Clarke
*abp1::ura4*^+^*ade6-704 leu1-32 ura4-D18 h*^+^	Y. Murakami
*cbh1::his3*^+^*ade6-704 leu1-32 ura4-D18 his3-D1 h*^+^	This study
*cbh2::sup3.5 ade6-704 leu1-32 ura4-D18 his3-D1 h*^-^	This study
*abp1::ura4*^+^*cbh2::sup3.5 ade6-704 leu1-32 ura4-D18 h*^-^	This study
*cbh1::his3*^+^*cbh2::sup3.5 ade6-704 leu1-32 ura4-D18 his3-D1 h*^-^	This study
*abp1::ura4*^+^*cbh1::his3*^+^*abp3::sup3.5 int. nmt81-abp1*^+^*ade6-704 leu1-32 ura4-D18 his3-D1 h*^-^	This study
*cdc23-M36 ade6-704 leu1-32 ura4-D18 his3-D1 h*^-^	P. Nurse
*cdc23-M36 ade6-704 leu1-32 ura4-D18 his3-D1 h*^+^	This study
*cdc23-M36 abp1::ura4*^+^*ade6-704 leu1-32 ura4-D18 h*^-^	This study
*cdc23-M36 cbh1::his3*^+^*ade6-704 leu1-32 ura4-D18 his3-D1 h*^-^	This study
*cdc23-M36 cbh2::sup3.5 ade6-704 leu1-32 ura4-D18 h*^-^	This study
*cdc23-M36 abp1::ura4*^+^*cbh2::sup3.5 ade6-704 leu1-32 ura4-D18 h*^-^	This study
*cdc23-M36 cbh1::his3*^+^*cbh2::sup3.5 ade6-704 leu1-32 ura4-D18 his3-D1 h*^-^	This study
*cdc18-K46 ura4-D18 h*^-^	P. Nurse
*cdc18-K46 abp1::ura4*^+^*ade6-704 leu1-32 ura4-D18 h*^-^	This study
*cdc20-P7 ade6-704 leu1-32 ura4-D18 h*^+^	P. Nurse
*cdc20-P7 abp1::ura4*^+^*ade6-704 leu1-32 ura4-D18 h*^-^	This study
*cdc20-M10 leu1-32 ura4-D18 h*^-^	P. Nurse
*cdc20-M10 abp1::ura4*^+^*ade6-704 leu1-32 ura4-D18 h*^-^	This study
*cdc21-M68 ade6-704 ura4-D18 h*^-^	P. Nurse
*cdc21-M68 abp1::ura4*^+^*ade6-704 leu1-32 ura4-D18 h*^-^	This study
*cdc30-2H4 ura4-D18 h*^-^	P. Nurse
*cdc30-2H4 abp1::ura4*^+^*ade6-704 leu1-32 ura4-D18 h*^-^	This study
*cut5-T401 leu1-32 ura4-D18 h*^-^	M. Yanagida
*cut5-T401 abp1::ura4*^+^*ade6-704 leu1-32 h*^-^	This study
*sna41-912 ade6-704 leu1-32 ura4-D18 h*^-^	S. Yamashita
*sna41-912 abp1::ura4*^+^*ade6-704 leu1-32 ura4-D18 h*^-^	This study
*orp2-2 leu1-32 ura4-D18 h*^-^	J. Leatherwood
*orp2-2 abp1::ura4*^+^*ade6-704 leu1-32 ura4-D18 h*^-^	This study
*orp2-7 leu1-32 ura4-D18 h*^-^	J. Leatherwood
*orp2-7 abp1::ura4*^+^*ade6-704 leu1-32 ura4-D18 h*^-^	This study
*cds1::ura4*^+ ^*ade6-704 leu1-32 ura4-D18 h*^-^	A. Carr

### Deletion of abp1^+ ^causes a cell cycle delay prior to initiation of DNA replication

**Figure 5:**
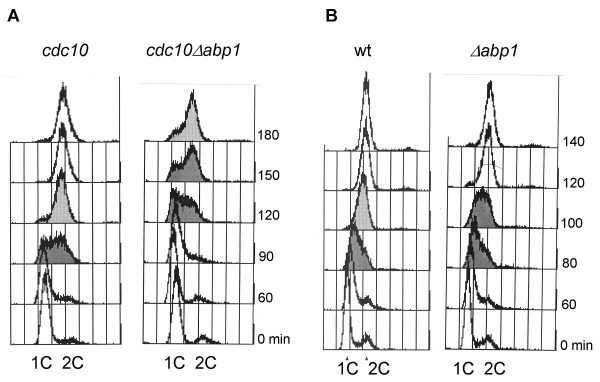
Cell cycle delay observed in *Δabp1 *strain is due to a DNA replication initiation defect. FACS analysis (DNA content) for *Δabp1 *strain (wild-type (wt) as control). **(A)**. After release from *cdc10-129 *block. **(B)**. After release from HU block.

The observation that Cdc23 (Mcm10), an essential replication factor, interacts genetically with Abp1 raised questions concerning the potential role of Abp1 in DNA replication. Although Abp1 was originally identified as a protein that binds to ARS (autonomously replicating sequences) *in vitro*, there was little evidence to support a role for this protein in DNA replication. Subsequently it was shown that Abp1 binds to centromere elements and that deletion of Abp1 results in a high frequency of chromosome mis-segregation. However, errors in DNA replication can also result in mis-segregation of chromosomes raising the possibility that Abp1 might have a role in S phase. To test whether cells deleted for Abp1 have any defects in their ability to replicate DNA, we monitored DNA content in cells following release from a G1 block. To arrest cells in G1, we constructed the double mutant *cdc10-129 Δabp1 *and shifted these cells to the restrictive temperature for *cdc10-129 *(36°C). Upon return to the permissive temperature, cells enter S phase synchronously and in the case of the *cdc10-129 *mutant alone, DNA replication is completed within two hours (Figure 5A, *cdc10-129*). However, in the double mutant, *cdc10-129 Δabp1*, S phase is delayed and there are still cells remaining in G1 150 mins following release (Figure 5A, *cdc10-129 Δabp1*). More importantly the persistence of the G1 peak 90 mins post-release suggests that the initiation events are specifically inhibited in cells lacking Abp1. Consistent with this idea, when *Δabp1 *cells are released from an early S phase arrest induced by hydroxyurea, rather than from a G1 block, no delay in DNA replication is observed suggesting that Abp1 is specifically required during DNA replication initiation (Figure 5B).

## Discussion

Initiation of DNA replication from chromatin-bound templates raises important questions concerning how DNA replication complexes gain access to origin DNA. It is likely that many auxiliary factors contribute to the assembly of both the pre-RC and its subsequent conversion to a fully functional replication fork on chromatin. Yeast genetics has provided a powerful tool to identify some of the key proteins that are required for assembly of these complexes. One of these proteins, called Cdc23 (Mcm10), is conserved from yeast to man, and is required for the early events of DNA replication initiation. It has been shown to interact directly with DNA polymerase alpha/primase and to stimulate primase activity *in vitro*. However, its precise role in S phase is still unclear. In an attempt to gain some insights into the function of Cdc23, we conducted a two-hybrid screen for Cdc23-interacting proteins. One of the proteins identified using this screen is Abp1, a protein that had previously been shown to bind to both ARS elements and centromeric DNA sequences. Although its primary function is thought be in chromosome segregation, we show that Abp1 may also have an additional role in DNA replication initiation.

First, Abp1 not only interacts with Cdc23 (Mcm10) protein in the two-hybrid analysis, but deletion of Abp1 lowers the restrictive temperature for *cdc23-M36*, consistent with its proposed role in DNA replication. Although two other proteins show significant homology to Abp1, deletion of either of these does not alter the phenotype of the *cdc23 *temperature-sensitive mutant, suggesting that Cdc23 (Mcm10) specifically interacts with Abp1. Furthermore, a more extensive genetic analysis of double mutants between *Δabp1 *and other replication mutants reveals that *Δabp1 *genetically interacts with both *orc2-7 *and *cdc30-2H4 *(encoding a mutant form of Orc1 and Orc2, respectively) and *cdc18-K46 (CDC6) *and *cdc21-M68 (MCM4) *(two mutants that are defective in the assembly of the pre-RC) providing additional evidence that Abp1 interacts with components of the replication initiation complex.

Our flow cytometry analysis suggests that *cdc23*^+ ^(*MCM10*) is required for DNA replication initiation. Previous studies also indicate that following a shift to the restrictive temperature of 36°C, *cdc23 *temperature-sensitive mutant cells arrest in early-S phase [32]. In our experiments a shift to the semi-permissive temperature of 30°C causes accumulation of cells with what appears to be a near 1C or G1-like DNA content suggesting that Cdc23, like its counterpart Mcm10 in budding yeast, is required for DNA replication initiation. This confirms that Abp1 interacts with a bona-fide DNA replication initiation protein.

Finally, we demonstrate, that cells deleted for Abp1 are significantly delayed from entering S phase following release from a G1 block. This delay is only observed following arrest in G1, and is not observed following release from an S phase arrest imposed by hydroxyurea. This implies that loss of Abp1 during S phase has no effect on replication kinetics, whilst loss of Abp1 prior to S phase can impede initiation.

It is still not clear how Abp1 might function to facilitate DNA replication initiation. One possibility is that Abp1, together with other chromatin-bound proteins, is important to remodel replication origins to allow access to the replication machinery. Alternatively, Abp1 might have a specific function in regulating origin firing in centromeric repeats. Interestingly, Abp1 has been shown to be important for heterochromatin modifications that are required for recruitment of Swi6 to chromatin where Swi6 is then responsible for nucleating formation of silent chromatin. So at least in this case, the presence of Abp1 can directly influence chromatin structure in relationship to DNA transcription. Note that the outer repeat region of the centromere where Abp1 binds was shown to be rich in ARS elements, and the ARSs were indeed functional as early replication origins [45]. It will be interesting to determine if Abp1, perhaps in conjunction with particular histone modifications and Swi6, may play a similar role in DNA replication.

## Methods

### Yeast strains and methods

*S. pombe *strains used in this study (Table 1) were derived from 972 h^- ^and 975 h^+ ^using standard genetic methods (Leupold, 1970). All media and growth conditions were as previously described (Moreno et al., 1991).

### Two-hybrid analysis

The *cdc23*^+ ^gene (bait) was cloned into pAS1 and co-transformed with pACT2 containing a *S. pombe *cDNA library (kindly provided by S. Elledge) into strain Y190 (*MAT****a ****gal4 gal80 his3 trp1-901 ade2-101 ura3-52 leu2-3,-112*, URA3::GAL-lacZ, LYS2::GAL(UAS)-HIS3 *cyh*^*r*^*2*). The histidine and β-gal assays were performed as previously described [46].

### Construction of Δcbh1 and Δcbh2 strains

Deletion strains for *cbh1*^+ ^and *cbh2*^+ ^were created using one-step gene replacement strategy. A 7.2 kb *XbaI/ApaI *DNA fragment containing the 1.6 kb *cbh1*^+ ^gene and its flanking regions was digested from the c9E9 and inserted into pBluescript-KS generating the plasmid pKS-*cbh1*^+^. A 6.4 kb *BglII/KpnI *genomic fragment containing the entire *cbh2*^+ ^coding region and its flanking regions was subcloned into pBluescript-KS from c14F5 generating pKS-*cbh2*^+^. To generate the plasmid Δ*cbh1:: his3*^+^, the pKS-*cbh*^+ ^recombinant plasmid was amplified in a *Dam *– *E. coli *strain, digested with *BclI*, which removed the *cbh1*^+ ^coding region almost entirely (from position 301), and replaced it with the *his3*^+ ^auxotrophic gene, obtained by digesting pAF1 with *BglII*. The resulting Δ*cbh1::his3*^+ ^plasmid was linearized and transformed into the diploid strain with the following genotype: *leu1-32/leu1-32 ade6-M210/ade6-M216 ura4-D18/ura4-D18 his3-D1/his3-D1 h*^+^*/h*^-^. Colonies that grew in agar plates lacking histidine were selected and the deletion confirmed by Southern Blot analysis. To construct the recombinant vector Δ*cbh2::sup3.5*^+^, the entire *cbh2*^+ ^ORF was deleted by inverse PCR from the pKS-*cbh2*^+ ^plasmid and replaced by the *sup3-5 *gene (0.5 kb). This plasmid was then transformed into the diploid strain with the following genotype: *leu1-32/leu1-32 ade6-704/ade6-M216 ura4-D18/ura4-D18 his3-D1/his3-D1 h*^+^*/h*^-^. Haploid colonies growing in the absence of adenine were selected. DNA was extracted, digested with *Hin*dIII the deleletion confirmed by Southern blot.

### Construction of Δabp1 nmt81-abp1^+^

A PstI/SacI fragment containing *nmt81-abp1*^+ ^was obtained from a partial digest of *pRep81-abp1*^+ ^and inserted into pJK-148 vector. After linearization at the *leu1 *locus with *Eco47III*, the linearized plasmid was transformed into the Δ*abp1::ura4*^+^*ade6-M216 leu1-32 ura4-D18 h*^- ^strain. Integration was confirmed by Southern blot analysis.

### Synthetic lethal genetic analysis

Exponentially growing cultures for each mutant analyzed were prepared. All cultures were adjusted to 10^7 ^cells/ml and a total of 6 serial 5-fold dilutions were spotted on YE plates and incubated at temperatures ranging from 25–36°C for 4 days.

### Hydroxyurea sensitivity

Cultures of each strain (wt, *Δcds1 *and *Δabp1*) were grown to an OD_595 _= 0.2 (log phase) and 5-fold serial dilutions of each strain were spot plated on YE plates containing 0, 2.5, 5 and 10 mM hydroxyurea. The first spot represents 10^7 ^cells plated. Plates were incubated at 32°C for 5 days. Assessment of viability for *wt, Δcds1*and *Δabp1 *at each HU concentration was performed.

### Cell synchronization experiments

To arrest cells in G1, *cdc10-129 *and *cdc10-129 Δabp1 *cells were incubated at 36°C for four hours. Cells were then returned to the permissive temperature of 25°C, collected at the indicated times and fixed for flow cytometry analysis (FACS). To block cells in S phase, wt and *Δabp1 *cells were incubated in 15 mM HU for 4 hours at 32°C. Cells were then pelleted, washed 3× with YE media to remove traces of hydroxyurea, and resuspended in YE media. Cells were then collected at the indicated times and prepared for FACS analysis.

### Flow cytometry

Cells were fixed in 70% ethanol, washed and resuspended in 50 mM sodium citrate, treated with 100 μg/ml RNase, followed by staining with 2 μM Sytox Green (Molecular Probes) or 0.5 ug/ml propidium iodine. Before processing, cells were sonicated, and DNA content was assayed using a Becton Dickson FacsSCAN.
